# Micro-to-Nanoscale Characterization of Femtosecond Laser Photo-Inscribed Microvoids

**DOI:** 10.3390/nano14141228

**Published:** 2024-07-20

**Authors:** Matilde Sosa, Maxime Cavillon, Thomas Blanchet, Gergely Nemeth, Ferenc Borondics, Guillaume Laffont, Matthieu Lancry

**Affiliations:** 1 Université Paris-Saclay, CEA, List, 91120 Palaiseau, France; matilde.sosamarti@cea.fr (M.S.); thomas.blanchet@cea.fr (T.B.); guillaume.laffont@cea.fr (G.L.); 2 Institut de Chimie Moléculaire et des Matériaux d’Orsay (ICMMO/SP2M/MAP), Université Paris-Saclay, CNRS, 91405 Orsay, France; maxime.cavillon@universite-paris-saclay.fr; 3 SMIS Beamline, SOLEIL Synchrotron, L’Orme des Merisiers, 91190 Saint Aubin, France; gergely.nemeth@synchrotron-soleil.fr (G.N.); ferenc.borondics@synchrotron-soleil.fr (F.B.)

**Keywords:** fiber Bragg gratings, femtosecond lasers, microvoids, high temperature

## Abstract

Fiber Bragg gratings are key components for optical fiber sensing applications in harsh environments. This paper investigates the structural and chemical characteristics of femtosecond laser photo-inscribed microvoids. These voids are at the base of type III fs-gratings consisting of a periodic array of microvoids inscribed at the core of an optical fiber. Using high-resolution techniques such as quantitative phase microscopy, electron transmission microscopy, and scattering-type scanning near-field IR optical microscopy, we examined the structure of the microvoids and the densified shells around them. We also investigated the high-temperature behavior of the voids, revealing their evolution in size and shape under step isochronal annealing conditions up to 1250 °C.

## 1. Introduction

In the field of optical sensing, fiber Bragg gratings (FBGs) are key components due to their small size, their immunity to electromagnetic interference, their spectral multiplexing capabilities, and their potential ability to withstand high pressures and temperatures [1]. More specifically, FBGs photo-inscribed using femtosecond lasers (fs-FBG) have emerged over recent decades since they offer selectivity in spatial and time domains [2,3]. They enable reliable and robust instrumentation to operate in various harsh environments at elevated temperatures, making them suitable for a wide variety of applications including in nuclear reactors [4], aeronautics [5], the oil and gas industry [6,7], the steel industry, and laser additive manufacturing [8].

According to the nature of the refractive index modification and the structural changes induced by fs-lasers, different types of gratings are identified [9,10,11]. Type I modifications, corresponding to an isotropic index change in the glass matrix, and mostly related to defect formation, densification, and fictive temperature change, present relatively low thermal stability (<600 °C for few hours in silica) [12]. Using femtosecond laser direct writing (FLDW) and tight focusing conditions, high temperatures (>1000–2000 °C) [13] and pressures (up to 200 GPa) [14,15] can eventually develop, leading to glass restructuring as a new composite material. These values are then clamped at higher energies, as reported in [14,16]. Such transformations, including type II and III permanent modifications, demonstrate remarkable thermal stability (1000 °C for at least 100s of hours) [17,18]. At temperatures around 1100 °C, the type II/III FBG reflectivity rapidly deteriorates and can even be lost during the annealing process [19].

While type II fs-FBGs are attributed to the formation of “light-forced organized” porous nanogratings inside the inscribed grating fringe, the type III fs-FBGs correspond to the formation of a periodic array of micro/nanovoids. Each void is created by focusing a single fs-laser pulse, modifying only a fraction of the core volume. The void size will depend on the focusing conditions and the laser parameters. Within this regime, focusing high-power fs-laser pulses in a small fraction of the volume will create a microcavity that is expected to be surrounded by a densified shell due to mass conservation. There are several investigations of the formation and the underlying structure of microvoids in bulk silica glasses [20,21,22] and in conventional telecom optical fibers [23,24]. The microvoids in bulk silica were studied by means of Raman spectroscopy, and Bressel et al. [20] confirmed a densification shell around the microcavity. Martinez et al. [23] also studied microvoids inscribed in optical fibers with quantitative phase microscopy (QPM); these authors showed that there is a negative and positive phase variation in the void and around it, respectively. In the same article, the authors discussed the possibility that densified matter and strain may be the cause of the positive variation around the voids, but mentioned that there are some limitations in the instrument resolution. In addition, Williams et al. [24] performed high-resolution measurements using scanning electron microscopy (SEM), reporting the size of the voids, between 100 nm and 400 nm, but failing to detect the densified shell under this type of observation. Moreover, Juodkazis et al. [25] demonstrated similar results, but these authors point out that the densified region is difficult to identify with the SEM technique.

All of these results provide indirect but strong evidence for the existence of a densified shell around the voids. However, studies at a nanoscale resolution are needed. Consequently, in this work, we will focus on high-resolution electron microscopy analysis and scattering-type scanning near-field optical microscopy (s-SNOM) in the infrared region, which will provide us with valuable insights for the analysis of microvoids and their densified shells at a nanometer scale. As previously mentioned, these void-based fs-FBGs can withstand temperatures as high as 1100 °C for several hours. Therefore, because of their increasing practical potential as sensors in harsh environments, we will also address the high-temperature stability of these microstructures and their behavior throughout a step isochronal annealing process up to 1250 °C.

## 2. Materials and Methods

Fs-FBGs were inscribed at the FemtoBragg platform (CEA List, Palaiseau, France), focusing femtosecond laser pulses inside the optical fiber core, using the point-by-point (PbP) direct writing technique. The femtosecond laser employed for this purpose was a Pharos laser (Light Conversion, Vilnius, Lithuania), operating at 515 nm with a pulse duration of 170 fs. The samples were fabricated inside a conventional SMF-28e single-mode optical fiber (GeO_2_-doped) from Corning (Corning, New York, NY, USA). The fiber, once stripped of its acrylate coating, was positioned on the platform, and the core was aligned by means of a 1.4 NA oil immersion microscope objective. The microvoids were inscribed one per pulse, side by side, with the possibility to change the energy of inscription in between each pulse. The pulse energy range was set between 30 nJ and 80 nJ. This range of energies was chosen since targeted applications are in the field of fs-FBGs, where a minimum energy level is necessary to obtain a high reflectivity, while a maximum energy level limits us in terms of the saturation of the Bragg peak obtained.

In order to characterize these voids, different types of micro-to-nano scale analysis were performed. First, we used the quantitative phase microscopy technique (QPM, from Iatia Vision Science) coupled with an optical microscope (BX60, Olympus Co., Tokyo, Japan). This technique acquired three optical images at different depths (±1 µm from the focus) using a piezo motor displacing the optical microscope objective along its axis. A digital quantitative phase image of the sample was produced by processing the three images via solving the intensity transport equation [26]. All QPM and optical microscopic images were acquired with a 1.35 NA ×100 oil immersion microscope (Olympus Co., Tokyo, Japan) objective.

A thin slice of microvoids was prepared with the focused ion beam (FIB) technique for subsequent observations under scanning electron transmission microscopy (STEM, ZEISS SUPRA 55 VP, Jena, Germany). Thus, we obtained a very thin sample of approx. 60 nm from the xz plane (x being the axis along the fiber and z the direction of the laser propagation). The observations were performed on a transmission electron microscope (FEI TITAN3 G2-ThermoFischer, Waltham, MA, USA) operating at 300 kV using different detectors, namely bright field (BF), annular dark field (ADF) and high-angle annular dark field (HAADF) detectors, in STEM mode. The HAADF mode was employed to detect chemical changes inside the sample, since the image intensity is mostly proportional to the atomic number of the elements, whereas the BF mode is more sensitive to the structural arrangement (e.g., crystallization) and strain inside the structure. The chemical distribution was studied using energy dispersive X-ray spectroscopy (EDS).

Moreover, experiments were carried out at Synchrotron SOLEIL (Saint Aubin, France) using a scattering-type scanning near-field optical microscopy (s-SNOM, IR-neaSCOPE, Attocube system AG, Haar, Germany) technique in the infrared (IR) spectral range. This technique couples the spatial resolution of atomic force microscopy (AFM) and the analytical capabilities of IR spectroscopy, resulting in an optical resolution of a few tens of nanometers [27,28]. This allowed the acquisition of the near-field amplitude and phase maps at 1130 cm^−1^, i.e., close to the resonance frequency of Si-O-Si asymmetric stretching vibration [29]. The amplitude and phase images of the AFM tip oscillation were also measured, providing information about the mechanical properties of the sample.

Lastly, the step isochronal thermal annealing process was performed to investigate the behavior of fs voids at high temperatures. The temperature was increased from room temperature up to 1250 °C, using smaller and smaller temperature steps as we approached the critical temperature around 1100 °C (20 °C, 850 °C, 1050 °C, 1100 °C, 1125 °C, 1150 °C, 1162 °C, 1175 °C, 1187 °C, 1200 °C, 1212 °C, 1225 °C, 1237 °C and 1250 °C). The sample stayed in the furnace for a duration of 30 min at each temperature step, followed by quenching in the air for subsequent analysis at room temperature (RT). Optical microscopy imaging and QPM analysis were performed on the fs microvoids after every temperature step. Note that the same sample was exposed to all set of temperature steps, performing measurements after each step and placing it back in the furnace for the next step.

## 3. Results

### 3.1. Characterizations at Room Temperature

**Figure 1:**
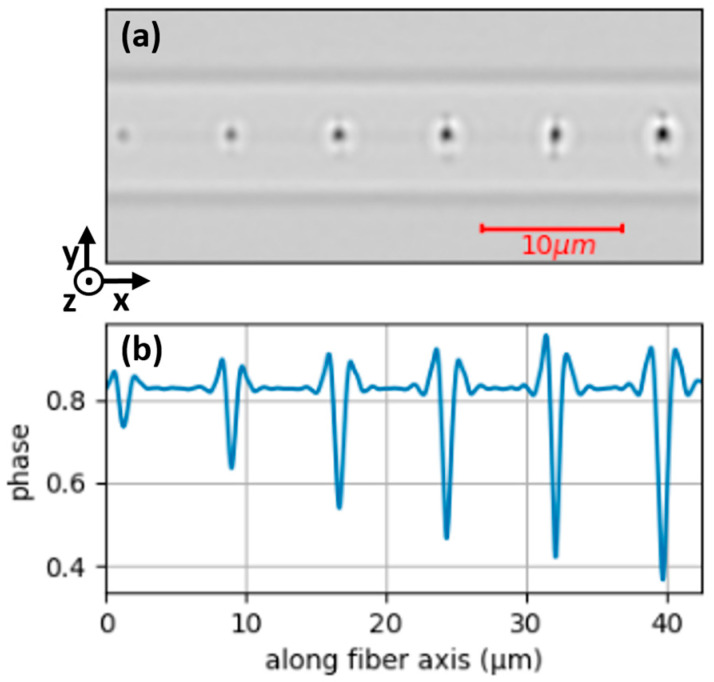
(**a**) Quantitative phase image of six microvoids using QPM, with energies of inscription varying from 30 nJ to 80 nJ with a step of 10 nJ (from left to right). This is a top-view of the optical fiber, with x representing the fiber axis and z representing the direction of the laser beam. The two horizontal lines, above and below the microvoids, are the interface between the core and the cladding of the optical fiber. (**b**) Phase profile (in rad) along the fiber core, passing through the center of the microvoids, averaged over a 1 µm wide window. Laser and focalization parameters: 515 nm, 170 fs, 1.4 NA, ×60.

Microvoids were first characterized at room temperature by means of QPM. Figure 1a shows a quantitative phase image of microvoids inscribed with different energies inside the optical fiber core. A high pass filter was applied in order to emphasize the observed phase contrasts at 550 nm. The microvoids were inscribed one by one, with six different fs-laser pulse energies varying from 30 nJ to 80 nJ, from left to right, with a 10 nJ step between them. We can observe in Figure 1 the relationship between the fs-laser pulse energy and the size of the microvoids. As the pulse energy increases, both the size of the microvoids and the phase image contrast increase. This can be quantitatively observed in Figure 1b, where the phase profile along the fiber axis shows a greater phase shift contrast, with negative values in the center of each void and positive ones around them. As the pulse energy increases, the size and the contrast also increase. Note that the refractive contrast is taken relative to the background.

In agreement with Martinez et al.’s [23] results, as seen in Figure 1a, a positive phase shift corresponding to the shell around the central void is observed. The increase in the phase at the sides of the void, and hence the increase in the refractive index, could be due to local densification. Indeed, through mass conservation, the regions surrounding the microvoids must be densified with respect to the pristine SiO_2_-GeO_2_ material. However, additional structural measurements, ideally at the nanoscale, would be necessary to confirm this suggestion. Thus, other microscopy techniques are required, such as electron microscopy or s-SNOM.

As a second step, from the FIB slice viewed under the STEM, we obtained some information regarding the true size and shape of the void, which could not be seen from the QPM images. From these measurements, we observed that the void (viewed from the xz plane perspective) has an elongated shape approximately 1 µm in length along the z axis and a height of 70 nm ± 5 nm along the x axis.

**Figure 2:**
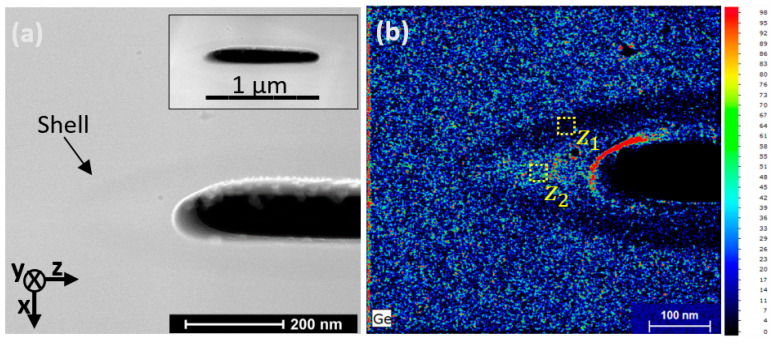
This is a xz side view of the core of the optical fiber. (**a**) HAADF-TEM observation of a microvoid written in the Ge-doped fiber core, with an energy of inscription of 60 nJ. Inset: entire microvoid observation (same characteristics). (**b**) EDS count map of germanium atoms (the scale is the number of hits on the detector). The Z_1_ and Z_2_ zones indicate the inside of the Ge-depleted area and outside it, respectively.

The thin white ring around the void, which can be seen in Figure 2a, resulting from the increase in the electronic emission efficiency, corresponds to an excess of gallium (Ga) deposition originating from the preparation of the sample with the FIB technique. This was verified by EDS. Moreover, in Figure 2a, a shell around the void is distinguishable. At this stage, we cannot confirm whether this contrast corresponds to the expected densified shell as discussed based on Figure 1, since it could originate from densification or a migration of chemical elements. For this purpose, the EDS analysis presented in Figure 2b, corresponding to a count map, reveals a slight depression in germanium (Ge) atoms. It should be noted that this chemical mapping of the Ge creates a “software artifact” at the void edge (red zone of the image). Therefore, we can conclude that there is a low concentration of Ge outside the void; however, nothing reliable can be established regarding the edge of the void. The concentration of GeO_2_ was measured in and outside the Ge-depleted zone (labeled Z_1_ and Z_2_ in Figure 2b, respectively), directly from the Ge/Si ratio. The depleted shell (Z_1_) exhibited a GeO_2_ content of ≈3.4 mol%, while outside it (Z_2_), the GeO_2_ content was found to be ≈5.2 mol%. Therefore, the GeO_2_ concentration fluctuation is low (at only 1.8 mol%), probably indicating that the index contrast observed within the shell is not due to an elemental migration. Indeed, a decrease in GeO_2_ around the void would lead to a decrease in the index, while from QPM, an increase in the index is observed instead.

**Figure 3:**
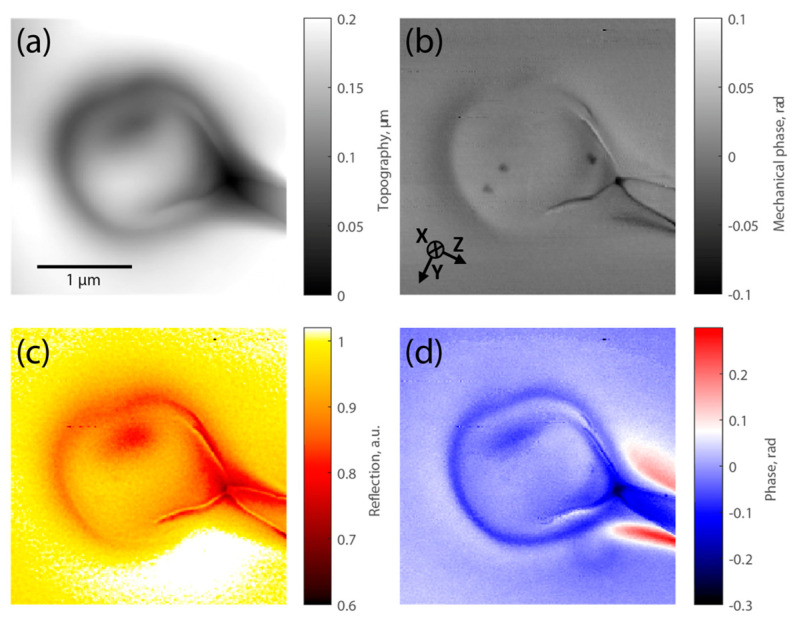
yz side views created by means of the AFM and s-SNOM techniques. These images show the microvoid at the fiber core (cross-sectional surface of a cleaved fiber), which has the same parameters as the TEM sample. (**a**) AFM topography. (**b**) AFM phase. (**c**) s-SNOM near-field amplitude map at 1130 cm^−1^. (**d**) s-SNOM near-field phase map at 1130 cm^−1^.

To further analyze these fs voids, we performed nanoscale s-SNOM measurements on a microvoid. In order to achieve this, we cleaved an array of periodic voids (a kind of FBG) written onto the fiber core. As the initiated fracture naturally propagates through the void, one can then observe the void cross-section from the perspective of the yz-plane, perpendicularly to the fiber axis. From this point, we were able to identify the shape of the void in this plane, as seen in Figure 3. Using this information and the previous measurements, we conclude that the void shape is an oblate spheroid, with a diameter of approximately 1 µm and a width of 70 nm ± 5 nm.

From these s-SNOM measurements, information about the presence of a densified shell from various aspects can also be obtained. The topology of the surface measured by AFM is shown in Figure 3a, and a dark shell corresponding to a negative surface u_xx_ is observed, i.e., a valley that is the mechanical signature of a densification occurring below the surface [30]. Figure 3b shows the AFM phase, which provides information on the mechanical properties of the sample. Here, the measured contrast may suggest a slight increase in the Young’s modulus, which could also be associated with a permanent densification [31]. The tail observed in Figure 3 is in the direction of the femtosecond laser beam propagation. This corresponds to the tail of laser tracks that is associated with stress and mechanical changes, as can be seen from the AFM map and has previously been reported in the literature [32].

Turning to the vibrational signature of SiO_2_-GeO_2_ glass at the nanoscale, the peak of the Si-O-T (T = Si or Ge) asymmetric stretching band denoted as $\nu_{as}^{Si-O-T}$ is located at approximately 1120 cm^−1^ [29], and our measurements were performed at 1130 cm^−1^ for that purpose. Thus, we can see that this phase difference is associated with a shift in the silica phonon band, either red or blue shift. From the near-field amplitude map at 1130 cm^−1^ as seen in Figure 3c, we can observe a higher amplitude of the backscatter signal surrounding the void. Figure 3d shows that the near-field phase map at 1130 cm^−1^ can complete this view. Interestingly, there is great phase contrast between the shell and the inside and outside of the void, revealing the presence of a shell owing to a low frequency shift in $\nu_{as}^{Si-O-T}$, which can then be associated with a permanent densification [29].

### 3.2. High-Temperature Behavior

**Figure 4:**
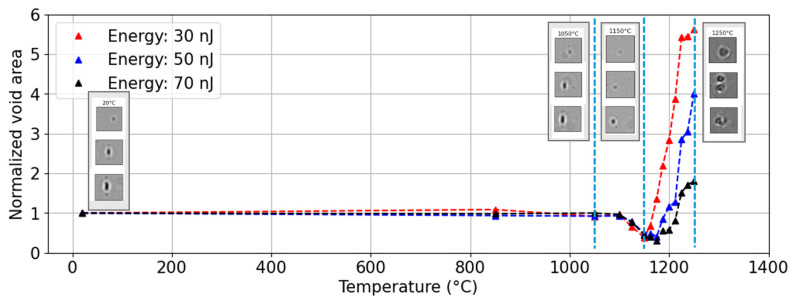
Normalized area evolution of three microvoids inscribed at 30 nJ, 50 nJ and 70 nJ over a 30 min step isochronal thermal annealing up to 1250 °C. Inset: Optical microscope images (top-view) after key temperature steps, namely at RT (≈20 °C), 1050 °C, 1150 °C, and 1250 °C.

In a last set of measurements, we investigated the thermal evolution of fs-written voids by means of an isochronal annealing process coupled with QPM measurements. Figure 4 provides an example of the normalized area of the voids as a function of the annealing temperature. Three different cases are illustrated, varying the energy of the laser direct writing. As observed from the inset (at key temperature steps, RT, 1050 °C, 1150 °C and 1250 °C), there is significant evolution of the microvoid shapes. First, the microvoids experience a significant reduction in size, to more than half of their original size, up to temperatures between 1150 °C and 1175 °C. All three then follow a similar behavior, although in different proportions, where the voids regrow and deform significantly up to 1250 °C. At 50 nJ, the void split into two or more sub-voids, which is also the case for other voids at other pulse energies. Moreover, at such high temperatures, the shell exhibiting a high phase around the void is still present.

## 4. Discussion

From the different measurement techniques used, we can coherently confirm the presence of a densified shell around the void. Indeed, while QPM reveals a positive phase contrast that may indicate either a densification or a migration of chemical species, from our STEM analysis, we can see that the latter is not the case. Additionally, from both strain relaxation measured by AFM and vibrational measurements from IR s-SNOM, we were able to provide clear evidence of localized densification. We also notice that this densified shell remains present at very high temperatures, even after the 30 min annealing step at 1250 °C, thus suggesting its formation under a high-pressure high-temperature mechanism.

Indeed, under strong focusing conditions, the process of fs-laser direct writing can trigger high levels of electron excitation. If the carrier plasma density goes beyond the critical value, the energy density can overcome the cohesion energy of the material. In short, the focal volume acts as a kind of microreactor with high pressures of up to 200 GPa [14] and temperatures higher than the glass softening temperature [33]. At short timescales, this creates a pressure difference between the center and periphery of the focal volume and ionic pressures can grow to values that exceed the mechanical strength of silica (~75 GPa [34]). This pressure difference induces a shockwave in the material towards the periphery of the plasma zone. This shockwave is followed by a rarefaction wave, accompanied by a relaxation wave, leading to a strong temperature increase. This results in a central area with low refractive index changes and possible fractures at high energy, surrounded by a hot compressed shell. The mass initially contained in this volume is pushed out and compressed and the glass material is quenched in a densified glass state (high fictive temperature—high fictive pressure glassy state), and hence it has a higher refractive index [15,25]. Micro-Raman studies also confirm the presence of a densified shell in SiO_2_ [35] and GeO_2_ [36] glasses, accompanied by molecular oxygen formation within the voids.

Let us now turn to the thermal stability. The effects of nanopore (type II regime) diameter as a function of temperature (from the Rayleigh–Plesset (R-P) equation) were investigated and discussed in [37]. As the initial nanopore diameter increases (from 50 nm to 150 nm), the annealing curves shift to higher temperatures (approx. 50 °C), resulting in a erasure temperature around 1100–1200 °C in SMF-28 [38]. Evolution at high temperatures also depends on the thermal history, such as pre/post-thermal treatments [19,39]. In the case of the microvoids, the shockwave not only induces a high fictive temperature but also a high fictive pressure shell, which might contribute to the observed higher thermal stability. Indeed, the higher the temperature during hot compression, the more stable the resulting densification is (e.g., a longer relaxation time τ [40]), associated with a narrower distribution of activation energy, and the more homogeneous the samples are [40]. This in turn influences the glass viscosity, enhancing the Young’s modulus but probably also the apparent surface tension (σ), resulting in highly stable voids. This view can be reinforced by the observed depletion of Ge atoms within voids and particularly in the densified shell that naturally results in a higher viscosity.

Examining the relaxation model discussed in [37] in detail, it is possible to simulate the erasure of nanopores during thermal annealing, thus establishing a preliminary picture of their thermal stability at high temperatures. This model considers the glass chemical composition, initial nanopore diameters, and surface energy values, with the viscous behavior of the glass being a major parameter with regard to the erasure at high temperatures. When the viscosity is approaching the glass transition temperature (T_g_) and even overtaking it, dR/dt (where R is the void radius) is always negative, i.e., the pore size decreases at least at the beginning, as occurs with nanopores. However, and counterintuitively, a regrowth of the voids follows the initial erasure. A possible explanation for this void regrowth could come from the thermal history itself, although additional experiments are needed to establish this link. During annealing and after each temperature step, the fiber is taken out of the furnace for QPM measurement. This process induces a strong thermal gradient between the nearly instantaneously cooled cladding outer periphery and the fiber core, which ultimately translates to a radial pressure gradient dP/dr. The latter can be estimated as dP/dr ≈ βBΔT/r_f_, with β, B, r_f_, and ΔT, respectively, being the volume coefficient of thermal expansion (silica: 5.5∙10^−7^ K^−1^), the bulk modulus (silica: 37 GPa), the fiber cladding radius (62.5 µm), and the temperature difference (typ. 1000 °C). This yields a dP/dr of ~1 MPa/µm. This pressure gradient, coupled with progressive glass softening associated with an annealing temperature increase, could possibly cause the voids to deform and expand. To qualitatively describe how such a pressure difference could yield to growth of the void, the simplified Rayleigh–Plesset equation [41] can be used,

(1) $$\frac{dR}{dt}=\frac{R\Delta P}{4\eta }-\frac{\sigma }{2\eta }$$

where *dR/dt* is the evolution of the void radius (*R*) as a function of time, Δ*P* is the pressure difference between the inside and the outside of the void (in Pa), σ is the surface tension (0.3 J/m²), and *η* is the glass viscosity (Pa·s). The first and second terms on the right-hand side of this equation would contribute positively and negatively to *dR*/*dt*. This means that the void growth is anticipated when Δ*P* > 2*σ/R*. Taking *R* as ~0.5 µm, this provides a Δ*P* of ~1 MPa, and this appears possible considering the previous values of thermal stress and considering the important pressure gradient over a few micrometers (10 s of MPa). One interesting aspect is that this regrowth is not observed for silica in the type II regime and under similar isochronal annealing experiments [37,38]. In fact, the type II regime is composed of nanopores with an *R* of ~0.01 µm. Consequently, this translates to a Δ*P* of ~60 MPa, which is too high considering stress gradients, and may explain why this effect is observed in the type III regime and not the type II regime. Additionally, when increasing the temperature from 1100 °C to 1250 °C in silica glass, the viscosity decreases by approximately an order of magnitude, and this could facilitate glass deformation. Additional experiments including modifications of the annealing conditions are needed to validate this hypothesis.

## 5. Conclusions

Microvoids inscribed using the direct writing technique in the core of a conventional telecom optical fiber using a fs-laser were studied using quantitative phase microscopy coupled with TEM, AFM, and nanoscale IR microscopy techniques. Based on the mechanical response and vibrational signature, the positive phase shell around the void was unambiguously attributed to local densification. High-temperature annealing experiments demonstrated the tendency of the voids to shrink from 1050 °C to ~1150 °C, followed by an increase in size and deformation above 1200 °C, while the densified shell remains present. Further studies are required to correlate this behavior with the spectral performances of fiber Bragg gratings or Fabry–Pérot-based sensors that are fabricated from these structures.

## Data Availability

Data underlying these results may be obtained from the authors upon reasonable request.
